# Pulmonary melioidosis complicating foreign body aspiration in a young adult

**DOI:** 10.1002/rcr2.819

**Published:** 2021-08-09

**Authors:** Mahesh Babu Vemuri, Archana Malik, Madhusmita Mohanty Mohapatra, Sujatha Sistla, Shahana Madan Purath, Sruthi Raj, Manju Rajaram

**Affiliations:** ^1^ Pulmonary Medicine Jawaharlal Institute of Postgraduate Medical Education and Research Puducherry India; ^2^ Microbiology Jawaharlal Institute of Postgraduate Medical Education and Research Puducherry India

**Keywords:** *Burkholderia pseudomallei*, melioidosis

## Abstract

Melioidosis is caused by an environmental Gram‐negative bacilli *Burkholderia pseudomallei*. Diabetes mellitus, occupational exposure to soil and water, pre‐existing renal diseases and thalassemia are significant independent risk factors for melioidosis. A 30‐year‐old male carpenter and smoker had a history of accidental aspiration of foreign body 2 months prior. On presentation, he had cough with expectoration and low‐grade intermittent fever for 1 month. His chest x‐ray displayed left lower zone consolidation with cavitation and presence of foreign body in the left lower lobe bronchus. Bronchoalveolar lavage inoculated onto 5% sheep blood agar and MacConkey agar grew *B*. *pseudomallei*. Melioidosis due to foreign body aspiration is rare. To the best of our knowledge, there have not been reports of melioidosis infection associated with foreign body inhalation. Hence, pulmonary melioidosis can be considered as a differential diagnosis in cases of foreign body with secondary infection even in immunocompetent host.

## Graphical abstract

Melioidosis is caused by an environmental Gram‐negative bacilli *Burkholderia pseudomallei*. Diabetes mellitus, occupational exposure to soil and water, pre‐existing renal diseases and thalassemia are significant independent risk factors for melioidosis. Herein, we report a case of melioidosis following foreign body aspiration and briefly review the literature on melioidosis.

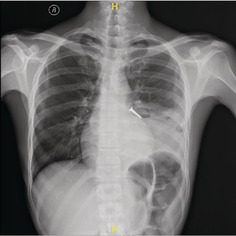

## INTRODUCTION

Melioidosis, caused by the environmental Gram‐negative bacilli *Burkholderia pseudomallei*, is classically characterized by pneumonia and multiple abscesses. It has diverse presentations and can affect any organ. Nearly 165,000 melioidosis cases are reported every year worldwide and its mortality rate is estimated to be 40%.^1^ It is of paramount public health importance in Southeast Asia and Australia, and is considered as a potential emerging infectious disease in many tropical developing countries.^2^ Eighty‐five cases of melioidosis have been reported from the Indian subcontinent from 1953 to 2016.^3^

Although commonly reported in diabetic patients, occupational exposure to soil and water, pre‐existing renal diseases and thalassemia are other significant independent risk factors for melioidosis.^4^ Melioidosis infection following foreign body aspiration has not been reported in the literature so far. Herein, we report a case of melioidosis following foreign body aspiration and briefly review the literature on melioidosis.

## CASE REPORT

**FIGURE 1:**
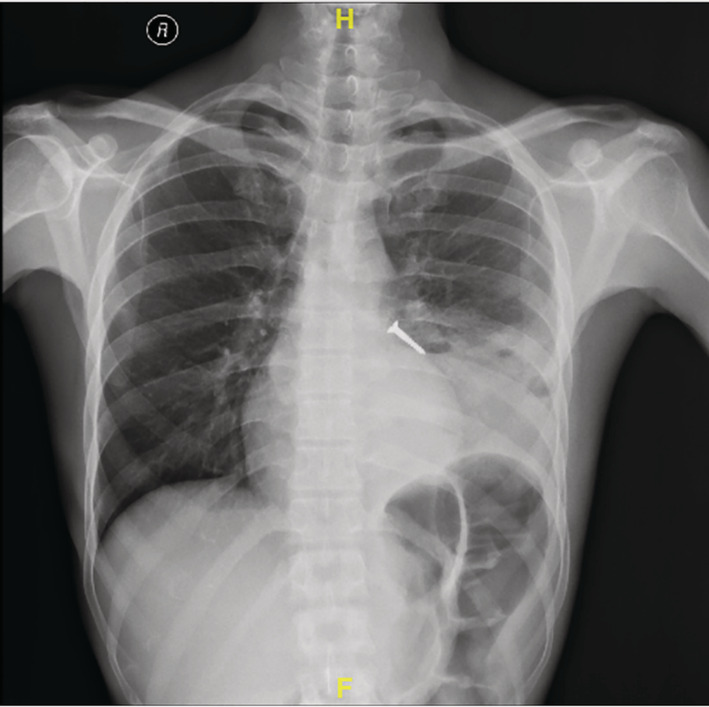
Chest x‐ray posteroanterior view showing left lower lobe consolidation with cavitation with foreign body in the left lower lobe bronchus

**FIGURE 2:**
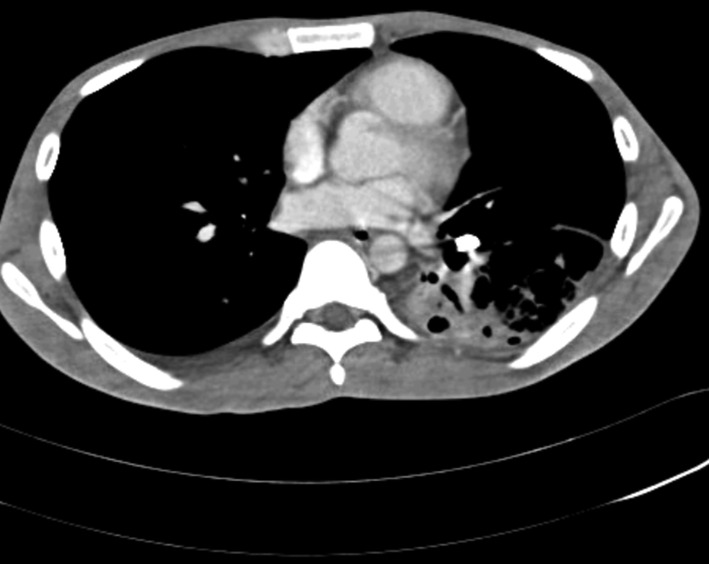
Contrast‐enhanced computed tomography showing a hyperdense foreign body (screw) lodged in the left lower lobe bronchus, with left lower lobe cavitating consolidation, centrilobular and tree‐in‐bud nodules noted in bilateral lung fields

A 30‐year‐old male, with diabetes and chronic alcohol consumption, presented with low‐grade fever and cough with expectoration for 1 month. Expectoration was yellow purulent and foul smelling, not associated with haemoptysis. He is a skilled carpenter. Two months back, he aspirated a foreign body while at work. As he was asymptomatic, he did not report to the hospital. There was no prior history of pulmonary tuberculosis and he denied anti‐tubercular therapy. He was conscious and oriented. Pulse rate was 90 beats/min, blood pressure 110/80 mm Hg, oxygen saturation 98% and respiratory rate was 20/min. He was febrile. On chest auscultation, bronchial breath sounds and fine pan‐inspiratory crackles were heard over the left lower chest. Chest radiograph was suggestive of left lower zone cavitating consolidation and a foreign body in the left lower lobe bronchus (Figure 1). Contrast enhanced computed tomography thorax showed a hyperdense foreign body, possibly a screw lodged in the left lower lobe bronchus, surrounding cavitating consolidation, centrilobular and tree‐in‐bud nodules in bilateral lung fields (Figure 2). A diagnosis of left lung cavitating consolidation secondary to foreign body aspiration was made.

**FIGURE 3:**
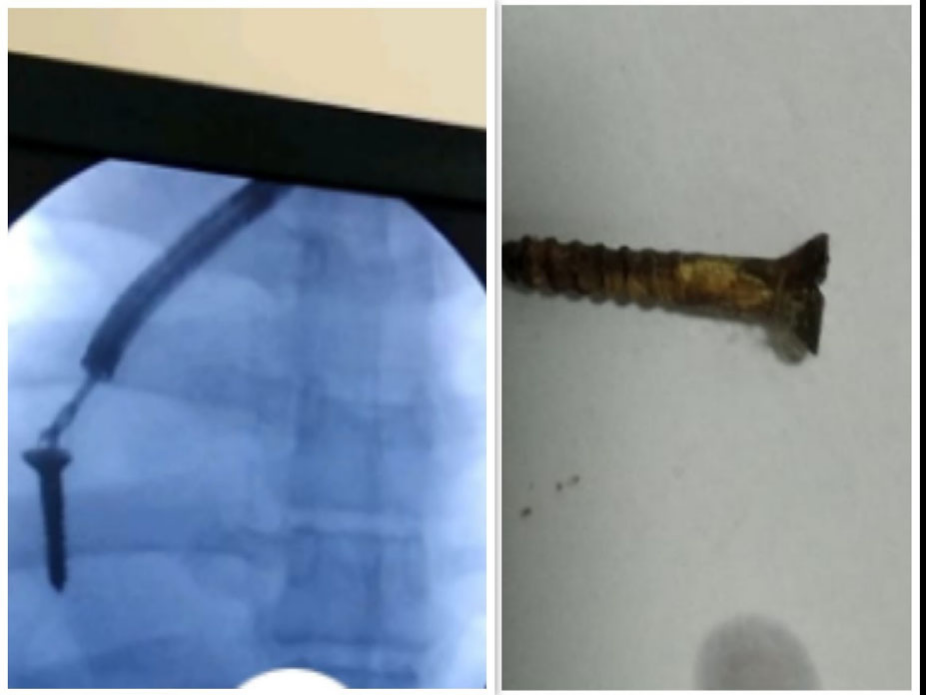
C‐arm‐guided removal of foreign body (screw)

**FIGURE 4:**
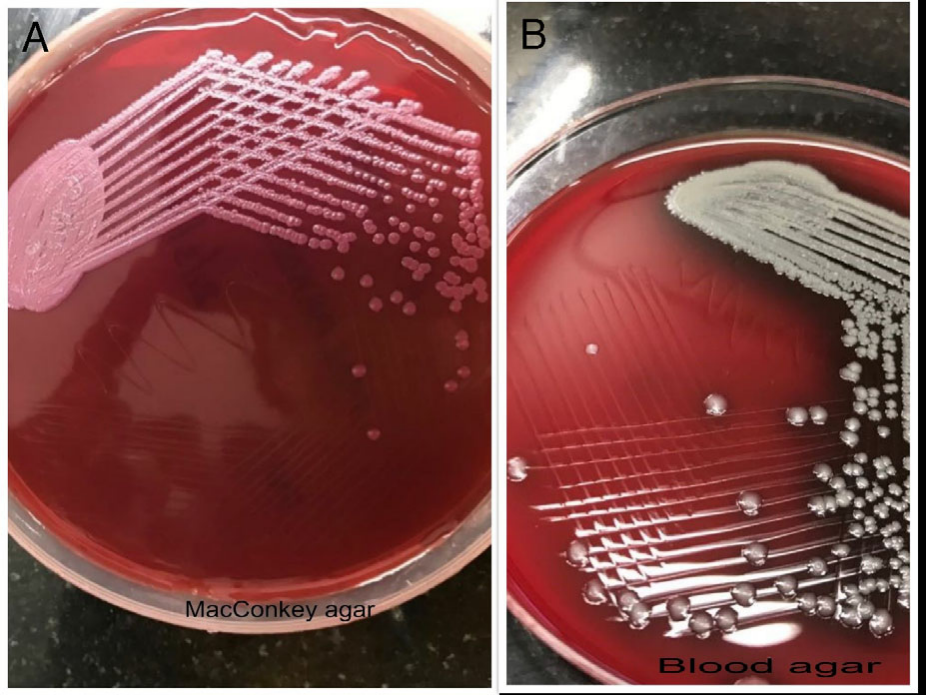
(A) MacConkey agar showing non‐lactose fermenting colonies. (B) Blood agar showing *Burkholderia pseudomallei* colonies

**FIGURE 5:**
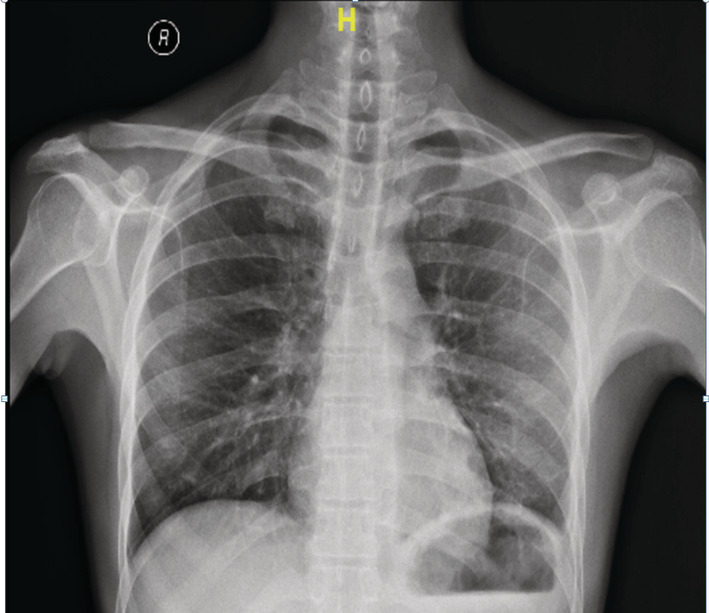
Radiological resolution of lesion

Blood and sputum cultures were sterile. Sputum acid fast bacilli smear and cartridge based nucleic acid amplification test were negative for mycobacteria. Mantoux test after 5 tuberculin units of tuberculin was negative. Bronchoscopic‐guided removal of foreign body was attempted by a multidisciplinary team comprising of pulmonologists and thoracic surgeons. A screw was retrieved (Figure 3). Bronchoalveolar lavage drawn from the left lingual and basal segments of lower lobe grew *B*. *pseudomallei* on 5% sheep blood agar and MacConkey agar. The organism was found to be sensitive to amikacin, cefoperazone, meropenem and doxycycline, and resistant to cotrimoxazole by the Kirby‐Bauer disc diffusion technique. They form non‐lactose fermenting colonies with metallic sheen on MacConkey agar. They appear dark pink or red after 4–7 days due to oxidation of lactose (Figure 4). There was no evidence of abscess formation in other organs. A final diagnosis of melioidosis with left lung cavitating consolidation of *B*. *pseudomallei* aetiology secondary to foreign body aspiration was further confirmed by antigen detection, antibody by indirect haemagglutination assay with a titre of 5120 and polymerase chain reaction targeting the Type III secretion system. The patient was treated with intravenous meropenem based on susceptibility data for 15 days. He was discharged with oral doxycycline for 3 months. The patient did not complete the oral eradication phase, is doing well and there is significant clinical and radiological improvement (Figure 5).

## DISCUSSION

Melioidosis is a great mimicker of tuberculosis. Similar to tuberculosis, it can remain latent in the body without active infection. It is endemic in Southeast Asia (Thailand, Malaysia, Vietnam, Cambodia, Laos and Myanmar) and northern Australia.^5^ Melioidosis can affect any organ of the body except hair and nails. *B*. *pseudomallei* is present in soil and surface water in endemic areas. In India, it is seldom seen. Diabetes mellitus, thalassemia disease, thalassemia traits, chronic kidney disease and excessive alcohol intake are well known risk factors for melioidosis.^6^ Our case is unique as it describes melioidosis in an immunocompetent host with no comorbidities.

Melioidosis is often transmitted by inhalation of contaminated dust or water droplets, percutaneous inoculation or ingestion of contaminated water. History of working in rice fields prior to the onset of infection almost triples the risk of acquiring melioidosis.^7^ Although our patient never worked in a rice field, he probably acquired the infection by inhalational route during foreign body aspiration.

Clinical manifestations of melioidosis range from localized infection to acute pneumonia and fulminant septicaemia. Based on a prospective study of 540 cases of melioidosis conducted by Currie BJ et al., the most common presentation was pneumonia, followed by genitourinary infection and skin infections.^5^ Consolidation is the most common radiological finding followed by nodular opacity which may enlarge, coalesce or cavitate.^5^

Definitive diagnosis of melioidosis can be achieved by isolation of *B*. *pseudomallei* from various clinical specimens, for example, blood in patients with septicaemia and pus aspirated from abscesses.^8^ In our case, *B*. *pseudomallei* grew from bronchoalveolar lavage culture. Current guidelines on melioidosis management recommend an initial intensive phase followed by an eradication phase.^9^ The duration of intensive phase is 10–14 days for uncomplicated infection or 4–6 weeks for persistent septic shock, deep‐seated organ abscesses, extensive lung disease, septic arthritis, osteomyelitis or neurological melioidosis.^9^ The 2014 Revised Royal Darwin Hospital guidelines recommend an intravenous intensive phase therapy with either ceftazidime if the patient is admitted in ward or meropenem if the patient is at the intensive care unit.^9^ Treatment of eradication phase is with trimethoprim sulphamethoxazole for at least 3 months. In our case, the isolate was resistant to cotrimoxazole, hence doxycycline was used. There was clinical and radiological improvement after treatment with doxycycline for 3 months.

Aspiration of foreign body is often complicated by pneumonia. The most common infection after foreign body aspiration occurs with mixed oropharyngeal flora, followed by *Streptococcus pneumoniae*, *Haemophilus influenzae* and *Moraxella catarrhalis*.^10^ However, in our case, foreign body aspiration was complicated by infection with *B*. *pseudomallei*.

In conclusion, *B*. *pseudomallei* is common in immunocompromised host but it is a diagnostic challenge in the immunocompetent host. Foreign body aspiration has not been reported as a risk factor for *B*. *pseudomallei* infection in the literature so far. We propose that pulmonary melioidosis should be considered among the differentials in case of foreign body aspiration resulting in infection, even in an immune competent host.

## CONFLICT OF INTEREST

None declared.

## ETHICS STATEMENT

Appropriate written informed consent was obtained for publication of this case report and accompanying images.
